# Current and Future Cancer Chemoprevention Strategies

**DOI:** 10.3390/pharmaceutics15051543

**Published:** 2023-05-19

**Authors:** Juan F. Santibanez, Victor H. Villar, Cesar Echeverria

**Affiliations:** 1 Group for Molecular Oncology, Institute for Medical Research, National Institute of the Republic of Serbia, University of Belgrade, Dr. Subotica 4, POB 102, 11129 Belgrade, Serbia; 2 Centro Integrativo de Biología y Química Aplicada (CIBQA), Universidad Bernardo O’Higgins, Santiago 8370993, Chile; 3 Cancer Research UK Beatson Institute, Garscube Estate, Switchback Road, Glasgow G61 1BD, UK; 4 Laboratory of Molecular Biology, Nanomedicine and Genomic, Faculty of Medicine, University of Atacama, Copiapo 1532502, Chile

Cancer is a leading cause of death worldwide, accounting for nearly 10 million deaths in 2020 and ranking as the second-leading cause of death in economically developed countries [1,2]. Due to the limited treatments available, preventive control strategies have facilitated new ways to create better therapeutic approaches to protect against cancer; in this sense, chemoprevention and its potential have generated many opportunities in recent decades.

Chemoprevention considers the use of natural or synthetic chemical agents to avoid, delay, or revoke cancer acting on the initial stages of tumor progression or reverse tumorigenesis to reduce tumors’ malignant tendencies and properties. At the cellular and molecular level, chemoprevention aims to control the dysfunctional protein activities during any of the initiation, promotion, and progression stages of cancer development [3].

This Special Issue examines the current and promising cancer chemopreventive agents that may provide ongoing evidence encouraging further research to develop novel drugs with potential clinical applications. Fourteen papers have been published: eight experimental and six comprehensive reviews dedicated to unveiling and understanding the synthetic or natural bioactive chemopreventive agents that aid in the mitigation of cancer development.

Lee et al. [4] addressed the in vitro antitumorigenic activity of arctiin, a lignan glycoside isolated from *Arctium lappa* L., on cervical cancer, the fourth most common malignancy diagnosed in women worldwide. Despite its low cytotoxic effect on HK-2, HeLa, and SiHa cell lines, arctiin was demonstrated to inhibit their cell migration and invasion in vitro. The author’s analysis to elucidate the underlying molecular mechanisms indicated that arctiin effectively reduced S100A4 expression, an inflammatory protein associated with cancer progression and metastasis, and downregulated PI3K activity. These results allow the authors to postulate upon the therapeutic potential of arctiin in cervical cancer.

Khuanphram et al. [5] investigated the chemopreventive activities of mixed sesame and orange seed extract (MSO) containing hesperidin and sesamin on the early stages of a diethylnitrosamine (DEN)-induced rat hepatocarcinogenesis model. The results demonstrated that the administration of MSO at high doses exhibited a robust inhibitory effect on preneoplastic lesion development in the liver along with reductions in the proliferation and induction of apoptosis. Moreover, MSO did not alter the activities of cytochrome P450 isozymes and detoxifying enzymes. Thereby, MSO showed malignant tumor prevention efficiency and might be safe when consumed with food or drugs due to the low risk of potential interactions with cancer drugs or natural extracts.

Lui et al. [6] demonstrated the preventive and therapeutic activities of flavokawain A (FKA), a kava chalcone isolated from the kava plant, by targeting the in vivo activated Ha-ras pathway in a non-muscle-invasive bladder cancer (NMIBC) mouse model that mimicked the human noninvasive papillary transitional urothelial cell carcinoma (UCC). FKA significantly reduced the tumor burden and increased the survival rate of UCC-bearing mice. At the same time, the cellular and molecular analyses indicated that dietary FKA effectively reduced cell proliferation along with apoptosis induction, determined by a tumor decrease in Ki67 and increase in p27 and survivin. Thus, FKA showed potential in preventing NMIBC recurrence and progression.

Drug repurposing has created new alternatives for cancer therapies and chemoprevention strategies. In this sense, the publication by Lauriola et al. [7] aimed to identify and characterize new compounds with lead-like properties that could modulate YAP-TEAD activity, a protein axis involved in cell proliferation and tumorigenesis, from a small library of 27 compounds. In particular, the selected drugs possessed structural features to act as protein–protein interaction (PPI) inhibitors that indirectly disrupt YAP/TAZ–TEAD interaction. The authors evaluated a drug panel on three colorectal and two ovarian cancer cell lines. The results indicated that seven compounds showed specific involvement in TEAD activity. In particular, a compound bearing a p-quinoid structure named IA5 effectively reduced YAP phosphorylation and YAP–TEAD complex transcriptional activity in colorectal cancer cell lines. Elucidating the IA5 molecular mechanism may potentially lead to the synthesis of more selective YAP-TEAD activity modulators with better antitumorigenic activities.

Signaling pathways are crucial in sensing and integrating extracellular cues to directly regulate these cellular processes or establish a transcriptional program to enable that adaptation. LKB1/AMPK1 and PI3K/mTORC1,2 are two signaling pathways which sense the energetic statuses of cells and nutrient availability, respectively. The relevance of modulating these pathways to prevent prostate cancer cells has been highlighted in this Special Issue. Sanchez et al. [8] showed that capsaicin, a phenolic compound in chili pepper, induces apoptotic cell death in prostate cancer cells. Using different approaches, the authors also demonstrated that this apoptotic effect is mediated by activating the LKB/AMPK pathway. In addition, although the authors proved that the capsaicin-induced activation of AMPK is independent of Ca^+2^, other cations may be involved, as the receptor potential cation channel subfamily V member 1 (TRPV1) prevented the activation of AMPK and the apoptotic phenotype. These results might be relevant to the prevention of prostate cancer; however, the link between the activation of AMPK, TRPV1, and apoptosis requires further studies.

The ability of cells to grow and proliferate is intrinsically linked with nutrient availability. PI3K/mTOR is a signaling pathway that senses nutrient availability, allowing cells to grow when the conditions are suitable. However, uncontrolled proliferation in cancer leads to nutrient deprivation conditions, in which cell proliferation is decoupled from nutrient availability. Indeed, mTORC1 is aberrantly activated in 80% of cancers, which supports this notion [9]. In this issue, Roudsari et al. [10] extensively reviewed the literature on the role of the PI3K/mTORC1 pathway in prostate cancer. In this review, the article authors described all the therapeutic agents that target PI3K/mTORC1 and the evidence supporting their efficacy. In addition, the authors argued that one of the drawbacks of inhibiting PI3K/mTORC1 is the activation of autophagy, a process that promotes survival. Thus, the authors proposed that combination therapy using PI3K/mTORC1 and autophagy inhibitors is likely a better therapeutic approach to treating prostate cancer.

Additionally, Santarelli et al. [11] determined the capacity of 3,4-Dihydroxyphenylethanol (DPE) to protect primary human colonic epithelial cells from Benzo[a]pyrene (B[a]P), classified as a Group I human carcinogen by the International Agency for Research on Cancer (IARC), and a common environmental and food contaminant, the increased concentration of which in food is linked to the cooking method used [12]. BPE effectively reduced the B[a]P-induced release of pro-inflammatory cytokines and chemokines. Moreover, this compound increased the rate of cell death while restoring autophagy and mitophagy cell activities and inhibiting mTOR and ERK1,2 signaling. These results demonstrated the potential use of DPE as a chemopreventive agent against colon carcinogenesis. Michalkova et al. [13] reported that a chalcone derivative (ZK-CH-11d) induced intrinsic apoptosis in an AMPK-dependent manner in breast cancer cells. Furthermore, the authors evaluated the role of autophagy, which is activated downstream of AMPK. However, the cell death was not rescued by the inhibition of autophagy, suggesting that autophagy is activated as a survival mechanism to counteract the effect of ZK-CH-11d.

The modulation of other signaling pathways that are aberrantly activated in cancer has also been evaluated in this Special Issue. Bertrand J et al. [14] synthesized purine analogs and assessed their effect on the activity of several protein kinases and the viability of cancer cells. Of note, the authors could define specific positions for chemical modification that led to the inhibition of specific kinases. Furthermore, some of the synthesized compounds exhibited cytotoxic effects and the ability to inhibit proteins downstream of Bcr-Abl and FLT3-ITD, highlighting the potential use of these compounds against leukemia. Accordingly, Ahsan et al. [15] reviewed the potential of chemopreventive targeting Nrf2, STAT3, and Src, critical proteins involved in multiple cancer initiation and progression pathways. The authors included relevant molecular mechanisms, biological information regarding cancer-associated protein signaling, and a wide-ranging analysis of chemopreventive compounds targeting Nrf2, STAT3, and Src. Due to the importance of these oncoproteins, this review provided literature data that may influence the development of natural/synthetic molecules and the design of new chemopreventive agents to reduce tumor-microenvironment-associated oxidative stress and inflammation and block their influence in cancer initiation and progression.

Transformed cells’ resistance to apoptosis is one of the hallmarks of cancer and is involved in uncontrolled tumor growth and metastasis. In this sense, combined therapies have emerged as promising strategies to fight cancer. Accordingly, Nor Hisham et al. [16] provided a complete overview of the potential use of Navitoclax (also known as ABT-263), an orally bioavailable small-molecule inhibitor of pro-survival Bcl-2 family proteins, combined with current chemotherapies. The authors based their article on the limited evidence of Navitoclax’s efficacy as a single agent in advanced and relapsed small-cell lung cancer patients. This review included preclinical evidence of the feasibility of combined approaches to improve navitoclax’s effectiveness and minimize potential side effects.

Gastric carcinoma is one of the fourth most common malignancies worldwide and remains the second-leading cause of cancer-related fatalities globally [17]. A considerable proportion of gastric carcinoma seems to be associated with *Helicobacter pylori* infection, a group 1 carcinogen according to the World Health Organization. Accordingly, Grosso and de Paz [18] addressed *Helicobacter pylori*’s biological and clinical features related to gastric cancer. They also compiled vital information on the current formulation used for infection eradication. Specifically, the authors summarized the recent advancements in developing amoxicillin-loaded gastroretentive drug delivery systems. This information may help develop novel and better-tuned materials against *Helicobacter pylori* gastric colonization.

Given the importance of aberrant epigenetic alterations in tumorigenesis, targeting epigenetics has become an attractive strategy for cancer therapy and chemoprevention. Ruzic et al. [19] provided an overall review compiling critical information on the abnormal activation of histone deacetylases (HDACs) in cancer. This review also focused on the first generation of classic small-molecule and dietary-derived HDAC inhibitors with demonstrated anticancer and chemopreventive activities. These included clinically approved HDAC inhibitors and selected phytochemicals with anti-HDAC activities. Additionally, a section was dedicated to future perspectives in the development of new HDAC inhibitors, including bifunctional compounds and proteolysis-targeting chimeras agents. In addition, the potential of combined strategies of HDAC inhibitors and current therapies in clinical trials were discussed.

Although in the early stages of cancer development, due to the small tumor mass, transformed cells do not entirely depend on a vascularization network, the generation of new blood vessels feeds the cells and greatly contributes to tumor growth and malignity [20]. Garcia-Caballero et al. [21] provided a comprehensive review article addressing the relevant literature on the importance of dietary-derived agents, emphasizing bioactive phytochemicals as angiopreventive compounds against urologic cancer. The authors effectively described the main physiopathological mechanisms of cancer angiogenesis and its crucial role in urological cancer. Moreover, the rationality of antiangiogenic therapies in cancer chemoprevention and the molecular targets of antiangiogenic phytochemicals were also discussed.

In this Special Issue, we hope to highlight the promising potential of natural products from diverse natural-source-inspired materials for various biomedical applications, such as cancer diagnosis and therapy. Naturally derived compounds with chemopreventive activity may also be safely included in anticancer strategies both in prevention and increasing cancer therapies with the final goal of reducing tumor burden and improving patients’ quality of life.
